# Comparison of infrared and solid-state ^13^C NMR spectroscopy for assessing soil organic carbon composition following hydrofluoric acid treatment

**DOI:** 10.1016/j.fmre.2024.12.011

**Published:** 2024-12-27

**Authors:** Chenglong Ye, Tongbin Zhu, Hui Guo, Jinquan Li, Ming Nie

**Affiliations:** a College of Resources and Environmental Sciences, Nanjing Agricultural University, Nanjing 210095, China; b Jiangsu Provincial Key Laboratory of Coastal Saline Soil Resources Utilization and Ecological Conservation, Nanjing Agricultural University, Nanjing 210095, China; c Key Laboratory of Karst Dynamics, MLR & Guangxi, Institute of Karst Geology, Chinese Academy of Geological Sciences, Guilin 541004, China; d Ministry of Education Key Laboratory for Biodiversity Science and Ecological Engineering, National Observations and Research Station for Wetland Ecosystems of the Yangtze Estuary, Institute of Biodiversity Science and Institute of Eco-Chongming, School of Life Sciences, Fudan University, Shanghai 200438, China

**Keywords:** Aliphatics, Aromatics, Carbohydrates, Silicate minerals, Spectroscopy

## Abstract

Although Fourier transform infrared (FTIR) spectroscopy is a common tool for analyzing the molecular composition of soil organic carbon (SOC), soil silicate minerals often interfere with the carbohydrate bands of FTIR spectra. Extraction with hydrofluoric acid (HF) to remove soil mineral phases is a routine treatment prior to SOC composition analysis using solid-state ^13^C nuclear magnetic resonance (NMR) spectroscopy. However, it is unclear whether HF pretreatment is suitable for FTIR spectroscopy and if the functional groups identified in the FTIR and NMR spectra are comparable after HF treatment. Utilizing a diverse array of soils from 47 upland forest sites across China, we investigated the correlations of organic molecular components quantified by FTIR spectroscopy before and after HF treatment with the widely used NMR spectra. Our results showed that HF treatment had little impact on SOC molecular composition as assessed by NMR spectroscopy. For HF-treated samples, carbohydrates (∼1,030 cm^−1^) and aliphatics (∼2,850 and ∼2,920 cm^−1^) measured by FTIR showed stronger correlations with NMR data (*r* = 0.75 and 0.69, respectively) compared with untreated samples (*r* = 0.35 and 0.29, respectively), likely due to effective mineral phase removal. Additionally, the SOC degradation index, expressed as carbohydrate-to-aliphatic ratio derived from FTIR spectra, correlated better with NMR results in HF-treated samples (*r* = 0.55) than in untreated samples (*r* = 0.19). However, both HF-treated and untreated samples showed limited correlation between aromatics measured by FTIR and NMR spectroscopy. These findings suggest that HF pretreatment under mild conditions (10% HF, room temperature) enhances the accuracy of FTIR analysis of carbohydrate and aliphatic components in SOC, highlighting its potential for rapid and large-scale analysis of these molecular constituents in mineral-rich soils across diverse geographic regions.

## Highlights

• HF treatment had little impact on SOC molecular composition in ^13^C NMR analysis.
• FTIR signals for carbohydrate C − O and aliphatic C − H were enhanced by HF treatment.
• HF treatment improved FTIR-^13^C NMR correlation for carbohydrates and aliphatics.

## Graphical abstract

**Figure:**
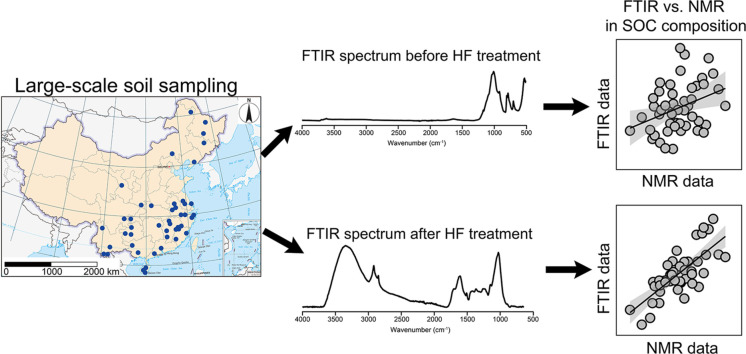

## 1 Introduction

Sequestering carbon (C) in soil organic matter has the potential to contribute to solving global climate change challenges [1]. Effectively managing soil organic C (SOC) stocks requires deep understanding of SOC formation, persistence, and function [2]. In general, SOC is understood to comprise a continuum of organic compounds encompassing varying sizes and degrees of decomposition derived from plant and microbial biomass [[3], [4], [5]]. Thus, molecular diversity is a potentially major mechanism driving SOC persistence [6,7], and knowledge of the chemical composition of SOC is needed for a better understanding of the mechanisms controlling SOC dynamics [8,9].

At present, solid-state ^13^C cross-polarization magic-angle-spinning (CPMAS) nuclear magnetic resonance (NMR) spectroscopy has been a popular and powerful analytical method for characterizing SOC chemistry [10], and can effectively distinguish SOC composition at the functional group level [11,12]. The primary structural assignments in NMR spectra include alkyl, *O*-alkyl, aromatic, and carbonyl groups [13]. To mitigate interference from soil minerals, hydrofluoric acid (HF) treatment has been a routine step to demineralize the soil sample, remove paramagnetic materials, and increase SOC concentration prior to analysis of SOC composition using solid-state ^13^C NMR spectroscopy [14,15]. However, characterization of SOC composition in large soil inventories is hampered by the fact that the ^13^C NMR technique is time consuming, expensive, and technically-demanding [16,17]. Hence, the utilization of the NMR technique is currently beyond the means of many laboratories.

Fourier transform infrared spectroscopy offers an attractive alternative for SOC chemical analysis due to its high-throughput, cost-effectiveness, and user-friendly nature, providing insights into the chemical bonds of organic components in soils [16,18]. Nonetheless, a great limitation of FTIR in SOC composition analysis stems from mineral interference, as soil minerals have absorption bands in the infrared region that can potentially overlap with SOC absorption bands. For example, Si−O bonds in silicates at around 1,030 cm^−1^ can obscure the C−O absorption bands of carbohydrates, making it difficult to accurately quantify these components [15,19,20]. Similarly, iron and aluminum oxides contribute broad low-frequency absorption features (below 700 cm^−1^), which interfere with the detection of organic functional groups in this range [21]. Despite these limitations, numerous studies have used FTIR spectroscopy to classify SOC functional groups without removing soil minerals [[22], [23], [24], [25], [26], [27], [28]]. In this context, multiple studies have attempted to predict the percentages of four broad classes of NMR-derived C types using FTIR spectra of untreated mineral soils combined with partial least-squares regression [29,30], but the prediction of aromatic C was frequently unsuccessful [29,31]. More importantly, it remains unclear whether FTIR-derived SOC functional groups from untreated soils can reliably correspond with those identified by NMR.

Given that organic signals in the FTIR spectra are superimposed on signals from minerals in soils, effective sample preparation emerges as a key strategy for overcoming this challenge. Theoretically, the application of FTIR analyses to HF-treated soils should facilitate direct peak integration, allowing for the quantification of the relative abundance of organic C groups [16]. Despite this potential, no study, to our knowledge, has assessed the suitability of HF pretreatment in FTIR spectroscopy and whether the resulting C functional groups align with the ^13^C NMR data. To address this gap, we selected a subset of soils collected from diverse forest ecosystems across China, showcasing a wide range of soil characteristics [32]. We hypothesized that the FTIR spectral signatures from HF-treated soils would exhibit stronger cross-correlation with the corresponding NMR chemical components compared to those from untreated soils. If there are robust correlations between C functional groups obtained from the two spectrometers through HF treatment, there is potential to develop more efficient and higher-throughput FTIR methods for assessing SOC composition, particularly in large-scale survey studies.

## 2 Materials and methods

### 2.1 Soil sampling

**Fig. 1:**
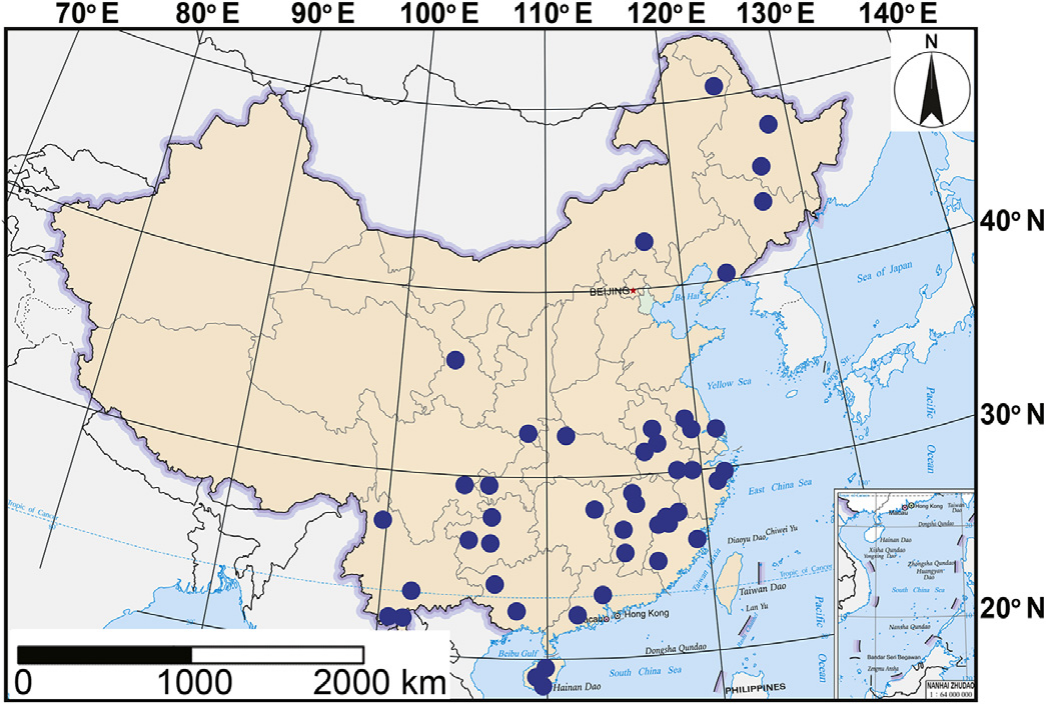
**Geographic distribution of soil sampling sites across China’s forest ecosystems.** The map is generated based on the standard map GS(2019)1696.

Soil samples were collected from 47 sites across China’s forests, representing a diverse array of site characteristics (Fig. 1). These sites span a wide range of latitudes (18.26° to 50.43° N) and longitudes (100.20° to 128.85° E), including most of the major forest types of tropical, subtropical, temperate, and boreal forests. The mean annual temperature (MAT) and mean annual precipitation (MAP) at these sites ranged from –2.2 to 25.0 °C and from 498 to 1,399 mm, respectively. The dominant species and soil types at different locations are listed in Table S1, with specific site information (e.g., geographic and climatic data) provided in Li et al. [32,33].

After removing the surface litter, surface mineral soils (0–10 cm) were collected from three random locations per site using a soil auger (diameter 7 cm). Then, these soil samples were thoroughly mixed to obtain one homogeneously composite sample at each site. All soil samples were immediately shipped back to the laboratory, and were passed through a 2-mm mesh after removal of visible stones and root fragments. Approximately 50 g of homogenized soil was then air-dried for determination of SOC molecular composition.

### 2.2 Sample preparation and ^13^C CPMAS NMR analyses

Air-dried soil samples were first washed by 3 M HCl to remove inorganic C and then ground to a fine powder using a Retsch MM400 mixer mill. Then, SOC concentration was determined using a CHNS analyzer (Elementar Vario Macro CUBE, Germany). In preparation for NMR analysis, acid-washed soil samples were treated with HF to remove paramagnetic compounds and mineral phases, leading to increased NMR sensitivity [14]. Briefly, approximately 5 g of finely ground soil was weighed into a 50 mL polyethylene centrifugation tube, saturated with 40 mL 10% HF, and then sealed and shaken for 8 h. After centrifugation, the supernatant was discarded appropriately, and the remaining slurry was then shaken with fresh HF solution again. After repeating the procedure five times, the remaining soil was washed with distilled water until the pH was above 5, and it was then oven-dried at 60 °C under a stream of dinitrogen gas. The concentrations of SOC after HF treatment were determined by the CHNS analyzer (Elementar Vario Macro CUBE, Germany).

Solid-state ^13^C CPMAS NMR spectra were acquired on these HF-treated soils using a 400 MHz Bruker AVANCE II spectrometer at room temperature (25 °C). In this method, soil sample was placed in a strong magnetic field and exposed to radiofrequency pulses. During cross-polarization, radiofrequency pulses are applied to the proton and C channels, transferring magnetization from protons to C for detection. The samples were then rotated at 54.74 °C during magic angle spinning, enhancing spectral resolution by averaging anisotropic interactions [10,34]. Briefly, weighed HF-treated samples (80−150 mg) with known C concentrations were placed in 4 mm diameter zirconium rotors with Kel-F caps to maximize the C mass and signal intensity. Spectra for all samples were acquired using a 12 kHz spinning speed, a ramp-CP contact time of 1 ms, and a recycle delay of 1 s. Between 10,000 and 20,000 scans were collected for each HF-treated sample with more scans collected for samples with lower C concentrations to improve the signal-to-noise ratio. Glycine was used as a standard to adjust the magic angle and as an external reference of ^13^C chemical shift. To assess the potential impacts of HF treatment on SOC composition, six untreated samples with relatively high SOC concentration (> 5%) were also selected for NMR analysis. Spectra for these untreated samples were recorded using the same operation conditions as the HF-treated samples and were acquired with over 30,000 scans.

**Fig. 2:**
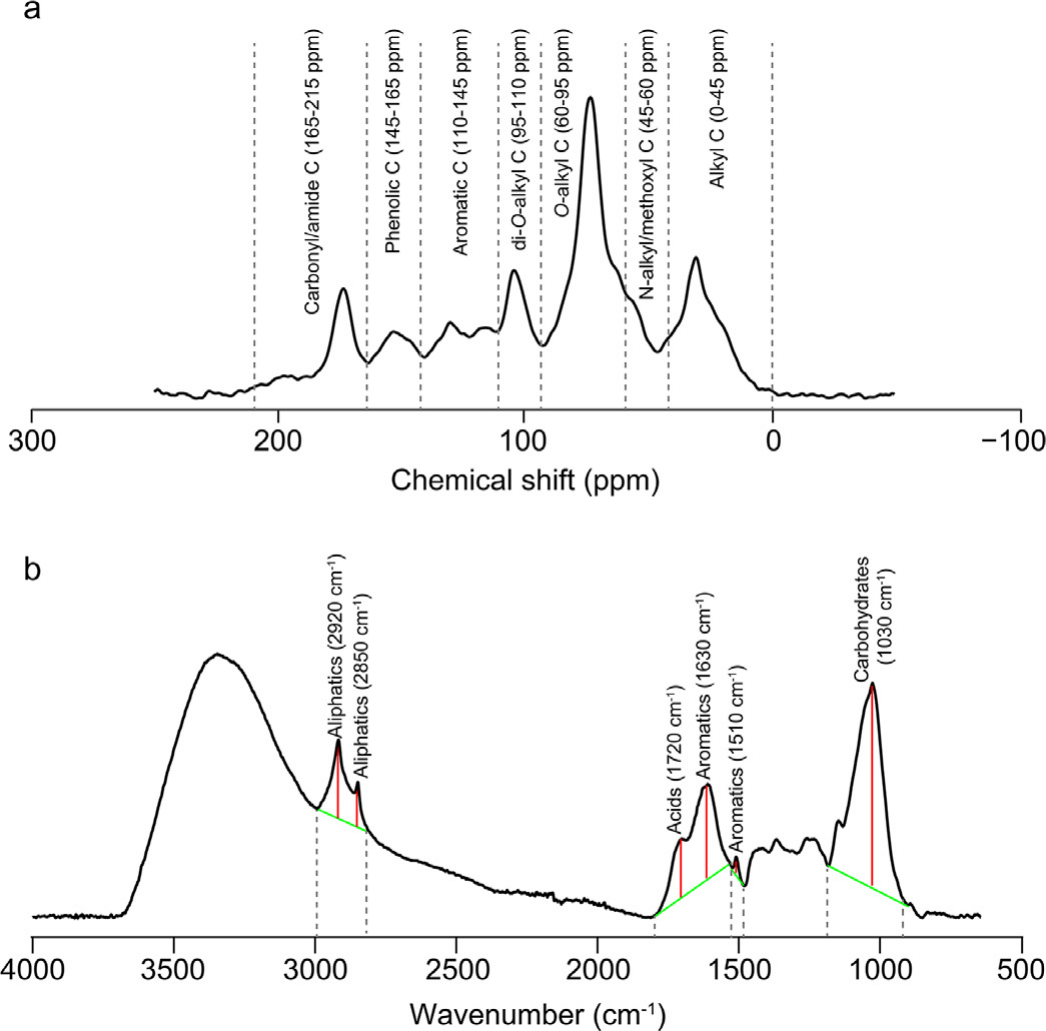
**Reference spectral regions and corresponding soil organic carbon types for solid-state ^13^C cross-polarization magic-angle-spinning (CPMAS) nuclear magnetic resonance (NMR) and Fourier transform infrared (FTIR) spectroscopy.** (a) The carbon types for ^13^C CPMAS NMR spectra are specified, with corresponding chemical shift ranges indicated in brackets and marked by vertical dotted lines. (b) Baseline corrections for FTIR spectroscopy peaks are shown with solid green diagonal lines for baselines, solid red vertical lines for baseline-corrected peak heights, and dotted gray vertical lines for baseline endpoints, with absorbance values normalized to the integrated area of the spectrum.

After baseline correction, the NMR spectra were divided into seven distinct chemical shift regions, each representing specific organic functional groups: 0−45 ppm (alkyl C), 45−60 ppm (N-alkyl/methoxyl C), 60−95 ppm (*O*-alkyl C), 95−110 ppm (di-*O*-alkyl C), 110−145 ppm (aromatic C), 145−165 ppm (phenolic C), and 165−215 ppm (carbonyl/amide C) (Fig. 2a) [9,35]. The relative contributions were quantified by integrating the signal intensities of different chemical shift regions using MestReNova 12.0.0 software, and expressed as percentage of the total area. A molecular mixing model was subsequently applied to the seven integrated spectral regions to estimate the relative abundances of six molecular SOC constituents: carbohydrates, proteins, lignin, lipids, carbonyls, and char. The C and N concentrations of HF-treated samples were incorporated as additional constraints in the molecular mixing model solutions [3,36].

### 2.3 FTIR analyses

In order to explore the interference of soil silicate minerals on the carbohydrate peak (∼1,030 cm^−1^) of FTIR spectra [37], attenuated total reflectance-Fourier transform infrared (ATR-FTIR) spectroscopy was used to analyze organic C composition in soil samples before and after HF treatment. Briefly, the fine soil powders were placed on the diamond crystal sample area of the benchtop FTIR spectrometer (Thermo Scientific Nicolet iS5), and the slip clutch pressure tower was lowered into position on the sample to keep the sample in place. The spectral data were collected across a range of 400 to 4,000 cm^−1^, and 64 scans were acquired for each of the spectra at the resolution of 4 cm^−1^. After each measurement, the crystal was cleaned with pure methyl alcohol, and background scanning was conducted to account for atmospheric changes. The spectra were converted from reflectance (R) to absorbance (log 1/R), smoothed, and then corrected for baseline using Thermo Scientific OMNIC software 8.2.0 (Thermo Fisher Scientific Inc., Madison, USA).

A custom R script was used to identify the exact location of peaks within predefined regions of the FTIR spectra (https://github.com/shodgkins/FTIRbaselines) [38]. Peak regions were determined individually for every sample, with baseline values defined by the minimum absorbance within the expected peak region (Fig. 2b). Notably, several key SOC functional groups exhibit characteristic absorbance peaks between wavenumbers 650 and 4,000 cm^−1^, allowing categorization based on peak maxima within this range. The specific FTIR absorption bands representative of these three functional groups were selected and quantified based on peak heights of these bands. The FTIR peak at ∼1,030 cm^−1^ was assigned to carbohydrate C−O stretching, the peaks at ∼1,510 cm^−1^ and ∼1,630 cm^−1^ were assigned to aromatic C=C stretching, and the peaks at ∼2,850 cm^−1^ and ∼2,920 cm^−1^ were assigned to the C−H stretching vibrations of aliphatic compounds, primarily originating from the C−H bond vibrations of the alkyl groups (hereafter referred to as aliphatic compounds) [23,26,38,39]. It is essential to acknowledge that the peaks at ∼1,510 cm^−1^ and ∼1,630 cm^−1^ can be substantially influenced by the presence of hydrated minerals. The overlapping absorptions may complicate the interpretation of these peaks, particularly in untreated soil samples where the resolution may be compromised. The removal effect of HF treatment on soil silicate minerals was evaluated based on the peak height at ∼780 cm^−1^ attributable to quartz and silicates. For each sample, the exact locations of the target FTIR peaks were determined, and baselines were constructed by connecting the endpoints adjacent to each peak. The peak height was then measured as the vertical distance from the top of the peak to the baseline. Finally, the baseline-corrected peak heights were normalized by dividing them by the total integrated area of the spectrum, yielding normalized corrected absorbance values (Fig. 2b). These calculations were performed using the R script provided by Hodgkins et al. [38].

### 2.4 Calculations and statistical analyses

Pearson correlation [cor() function in the R base] was calculated between individual SOC functional groups (carbohydrates, aliphatics, and aromatics) obtained from FTIR spectra before and after treating soil samples with HF, and their corresponding groups (carbohydrates, lipids, and lignin) obtained from NMR spectra. Additionally, correlation analysis was conducted for SOC degradation parameters (i.e., carbohydrate-to-aromatic ratio and carbohydrate-to-aliphatic ratio) calculated from the FTIR and NMR spectra. In this study, the selected level of significance was set at 5% (*P* < 0.05). All data analyses were performed using *R* (version 4.3.2).

## 3 Results

### 3.1 Effect of HF treatment on SOC concentrations and silicates

**Fig. 3:**
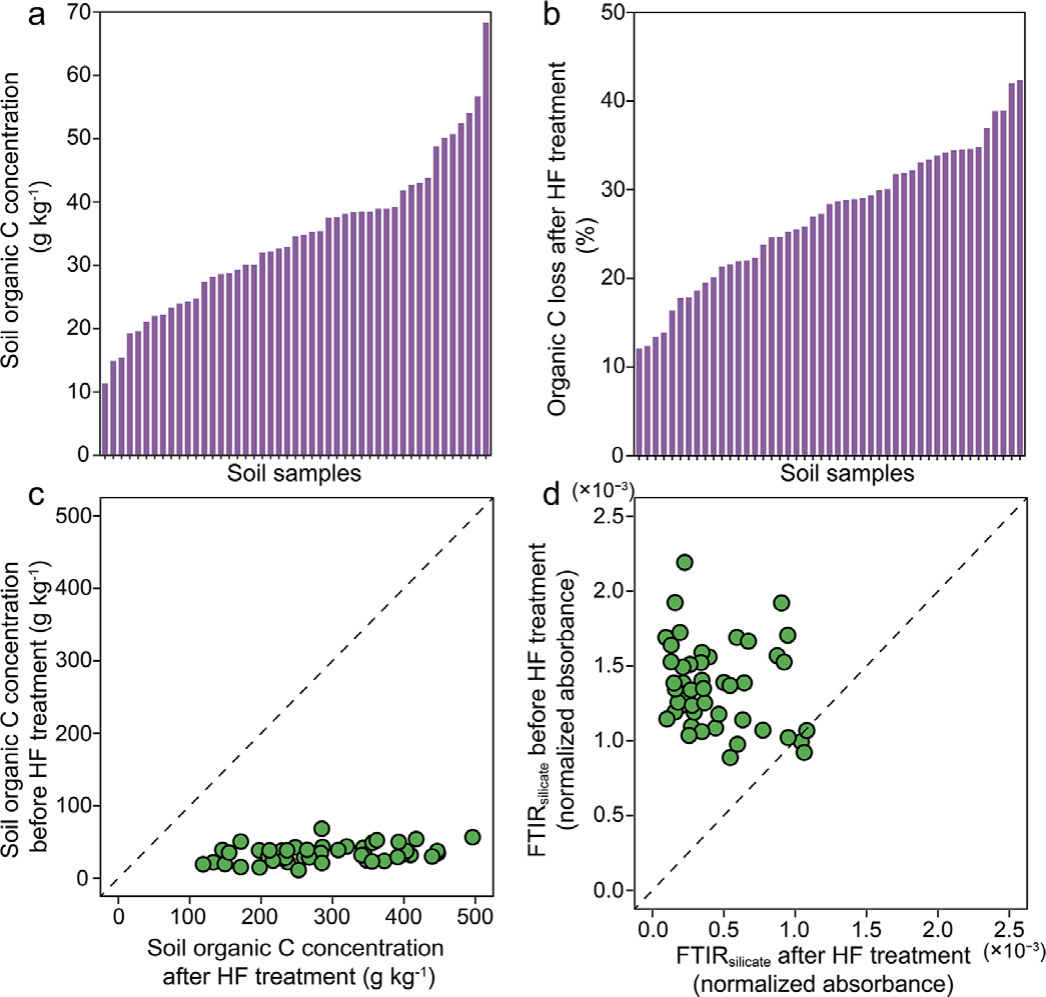
**Effects of hydrofluoric acid (HF) treatment on soil organic carbon and mineral composition, as indicated by silicates.** (a) Distribution of soil organic carbon concentrations across soil samples, with values presented in ascending order. (b) Soil organic carbon loss following HF treatment, with values presented in ascending order. (c) Comparison of soil organic carbon concentrations before and after soil samples treated with HF. (d) Normalized absorbance of silicates at 780 cm^−1^ obtained from Fourier transform infrared spectra before and after soil samples treated with HF. The normalized absorbance of silicates is calculated by adjusting the absorbance values relative to the integrated area of the spectrum. The dotted lines represent the 1:1 relationship.

The SOC concentrations in the untreated samples varied between 11 and 68 g kg^−1^ (Fig. 3a). Following HF treatment, there was an average reduction of SOC by approximately 27%, with losses ranging from 12% to 42% (Fig. 3b). However, HF treatment also led to a significant increase in SOC concentrations, ranging from 119 to 508 g kg^−1^ (Fig. 3c). Concurrently, FTIR spectra revealed that HF treatment resulted in a significant reduction in peak height of quartz (∼780 cm^−1^), indicating a substantial mass loss of soil minerals after HF treatment (Fig. 3d).

### 3.2 Effect of HF treatment on SOC composition derived from NMR spectra

Following HF treatment, distinct ^13^C CPMAS NMR spectra were observed across the diverse soils (Fig. S1). On average, carbohydrate, lignin, lipid, and protein constituted 24.9%, 23.9%, 20.5% and 20.3% of SOC, respectively (Fig. S2). Although HF treatment resulted in slight reductions in carbohydrate and protein, with minor increases in lipid and lignin, linear relationships for these molecular components before and after HF treatment remained largely consistent (*r* values 0.97–0.99; Fig. S3), indicating that SOC composition derived from NMR spectra can be reliably compared across diverse soils due to their uniform response to HF treatment.

### 3.3 Comparison of SOC composition derived from FTIR and NMR spectra

**Fig. 4:**
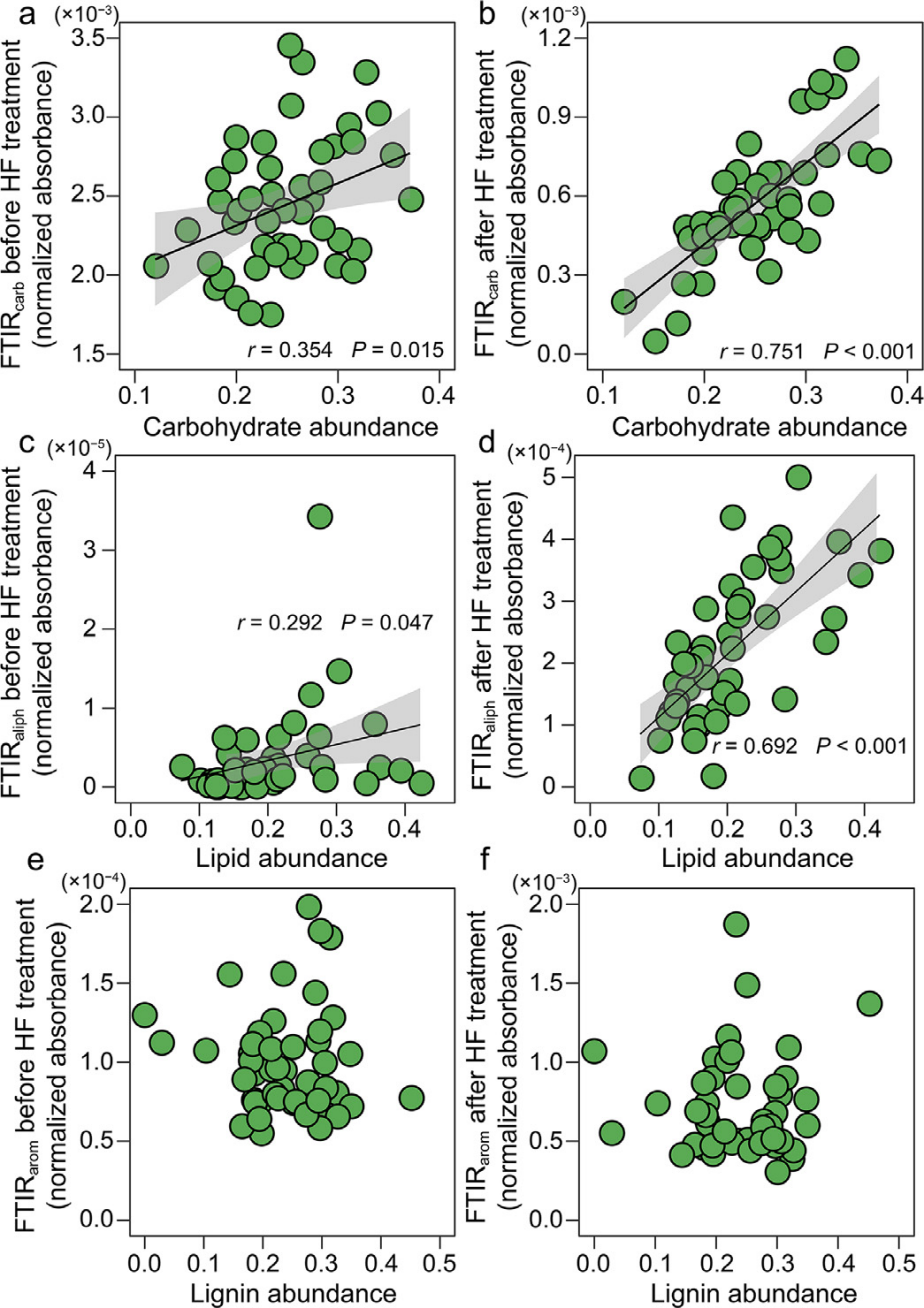
**Correlations of carbohydrate (a, b), aliphatic (c, d) and aromatic (e, f) groups from Fourier transform infrared (FTIR) spectra before and after treating soil samples with hydrofluoric acid (HF) compared to the corresponding soil carbon constituents from ^13^C cross-polarization magic-angle-spinning (CPMAS) nuclear magnetic resonance (NMR) spectra.** FTIR_carb_, FTIR_aliph_, and FTIR_arom_ represent area-normalized absorbance values corresponding to carbohydrate, aliphatic, and aromatic groups, respectively. The abundances of carbohydrate, lipid, and lignin were determined by applying a molecular mixing model based on the peak areas obtained from ^13^C CPMAS NMR. The shaded areas represent the 95% confidence intervals.

**Fig. 5:**
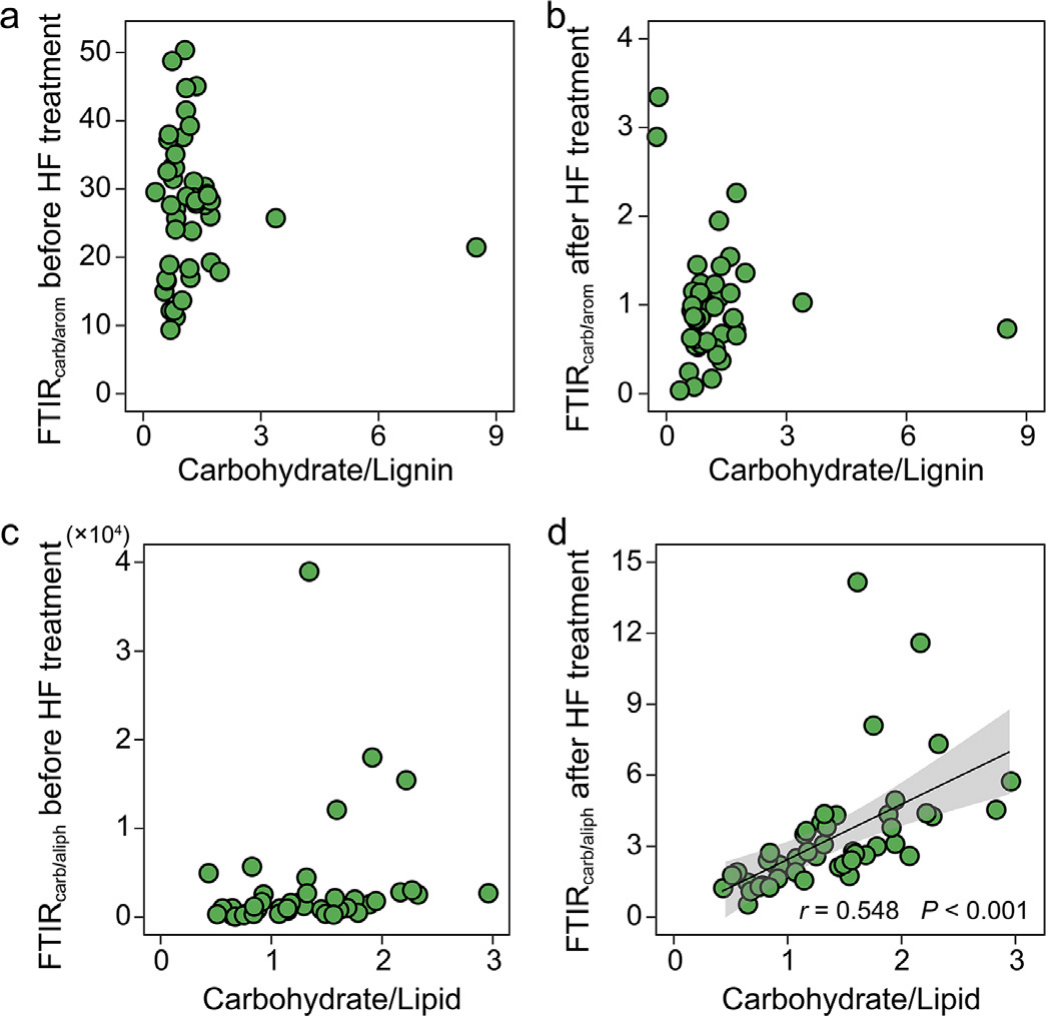
**Correlations of carbohydrate-to-aromatic ratio (a, b) and carbohydrate-to-aliphatic ratio (c, d) from Fourier transform infrared (FTIR) spectra before and after treating soil samples with hydrofluoric acid (HF) compared to the corresponding ratios from ^13^C cross-polarization magic-angle-spinning nuclear magnetic resonance spectra.** Carbohydrate-to-aromatic and carbohydrate-to-aliphatic ratios were calculated from area-normalized absorbance values obtained from FTIR spectra. The shaded areas represent the 95% confidence intervals.

The FTIR spectra of HF-treated samples showed substantial variability in peak intensities for aromatics (∼1,510 cm^−1^ and ∼1,630 cm^−1^) and aliphatics (∼2,850 cm^−1^ and ∼2,920 cm^−1^) across diverse soils (Fig. S4). In contrast, untreated samples displayed barely discernible peaks of these functional groups (Fig. S5). In comparison with untreated samples, carbohydrates and aliphatics in HF-treated soils measured by FTIR showed stronger correlations with corresponding C groups measured by NMR (Fig. 4a−d). However, regardless of HF treatment, aromatics exhibited a lack of significant correlation between FTIR and NMR measurements (Fig. 4e, f). Furthermore, the SOC degradation parameter, i.e., carbohydrate-to-aromatic ratio, did not exhibit a significant correlation between FTIR and NMR data, irrespective of HF treatment (Fig. 5a, b). In contrast, another SOC degradation parameter represented by the carbohydrate-to-aliphatic ratio showed a significantly enhanced correlation with the corresponding NMR data in HF-treated samples compared to untreated samples (Fig. 5c, d).

## 4 Discussion

In this study, we examined how HF pre-treatment of mineral soils influenced the accuracy of FTIR spectroscopy in analyzing SOC functional groups across diverse soil orders at the regional scale. Consistent with our expectation, FTIR analyses of HF-treated soils enhanced the comparability of spectra by reducing band overlap issues, thereby facilitating a more accurate assessment of SOC functional groups, specifically carbohydrates and aliphatics, as characterized by ^13^C CPMAS NMR spectra. These findings suggest that FTIR analyses of HF-treated soils could serve as a rapid and cost-effective method to provide valuable, albeit partial, insights into the chemical composition of SOC.

A significant improvement in the quality of solid ^13^C NMR spectra with minimal alteration has been reported with the use of HF treatment, particularly for mineral soils [14,15]. Thus, correlating the widely used NMR spectra with those of FTIR spectra from both untreated and HF-treated soils provides a means to evaluate the effectiveness of HF pretreatment in FTIR spectroscopy for analyzing C functional groups. In our study, compared with untreated samples, the normalized absorbance of carbohydrate peaks in HF-treated soils as measured by FTIR showed stronger correlations with the corresponding C group measured by NMR (Fig. 4a, b), suggesting that HF treatment largely reduces the interference from soil silicates, which generate a prominent FTIR peak overlapping with the carbohydrate signal (∼1,030 cm^−1^) [15,38]. Our study also found that HF treatment greatly improved the spectroscopic resolution of aliphatics, as indicated by the distinct peaks emerging at ∼2,850 cm^−1^ and ∼2,920 cm^−1^ (Fig. S4). Furthermore, the enhanced correlation between aliphatics obtained from the FTIR and NMR spectrometers was observed following HF treatment (Fig. 4c, d). Together, these results highlight the potential of HF demineralization to improve and broaden FTIR characterization of carbohydrates and aliphatics in mineral soils.

In contrast, insignificant correlation between aromatic components measured by FTIR and NMR spectroscopy following HF treatment was observed (Fig. 4e, f), indicating that HF treatment may hinder the FTIR characterization of aromatics. Several factors may contribute to this lack of correlation. First, there is an inherent overlap of peaks associated with aromatic and amide groups in FTIR spectra [40,41]. Additionally, aromatic compounds are known to be preferentially adsorbed by reactive metal minerals (i.e., solid iron phases extracted by citrate-bicarbonate-dithionite [42]) present in the soil [43]. Since HF solutions could effectively remove paramagnetic minerals from the soil matrix [14], it can be hypothesized that HF treatment facilitates the removal of aromatic compounds, further complicating spectral interpretation. Second, incomplete mineral removal may have occurred, as certain soil mineral components containing hydroxyl groups dominate the spectral regions at ∼1,510 cm^−1^ and ∼1,630 cm^−1^ due to their high molar absorptivity [20]. This dominance could further complicate spectral interpretation and affect the detection of aromatic signals. Third, a significant linear relationship was observed for both carbohydrates and aliphatics measured by FTIR before and after HF treatment (Fig. S6a, b), whereas such a relationship was absent for aromatics (Fig. S6c). A similar lack of correlation was also observed for carboxyl groups at 1,720 cm^−1^ (Fig. S6d). Although HF treatment increased peak intensities in the regions at 1,500–1,800 cm^−1^, mineral interference and SOC loss during demineralization are likely to introduce variability that complicates the accurate quantification of aromatic components.

The FTIR data also enable the quantification of SOC degradation indices, such as the ratios of carbohydrate-to-aromatic and carbohydrate-to-aliphatic, through the calculation of normalized absorbance ratios of the corresponding compounds [38]. In our study, HF treatment increased the correlation between FTIR and NMR data for the carbohydrate-to-aliphatic ratio (Fig. 5c, d). However, HF treatment did not improve the correlation for the carbohydrate-to-aromatic ratio (Fig. 5a, b). This difference may result from overlapping spectral features in the aromatic region and mineral interference, which can complicate the accurate detection of aromatic signals in FTIR spectra [41]. These results indicate that HF pretreatment can enhance the accuracy of SOC quality assessment based on the carbohydrate-to-aliphatic ratio when using FTIR spectroscopy.

While our findings highlight the potential of HF pretreatment to enhance the chemical characterization of SOC using FTIR spectroscopy, several limitations remain. First, HF treatment did not improve the accuracy of FTIR measurements for aromatic C, likely due to overlapping spectral peaks from amide compounds and the preferential binding of aromatic compounds to reactive minerals that HF removes [40]. Second, HF is highly toxic and poses significant health risks, requiring careful handling and specialized equipment [41]. These safety concerns restrict the broader use of HF in routine soil analyses, emphasizing the need for alternative methods that minimize health hazards while ensuring analytical accuracy. Third, while HF pretreatment improved the identification of carbohydrates and lipids, which likely account for < 50% of the total SOC, this suggests that the method may not fully capture the complexity of SOC, particularly in soils with a significant concentration of aromatic compounds or proteins. Thus, developing approaches to improve the correlation between FTIR and NMR measurements of SOC components, particularly aromatic C, and to reduce HF use would support broader ecological and biogeochemical applications while improving the accessibility and affordability of SOC analysis for large-scale studies.

## 5 Conclusion

Our study demonstrates that HF pretreatment of mineral soils enhances the performance of FTIR spectroscopy in characterizing carbohydrate C−O (∼1,030 cm^−1^) and aliphatic C−H bands (∼2,850 and ∼2,920 cm^−1^), as validated by correlation analyses of corresponding SOC groups obtained from ^13^C CPMAS NMR data. Furthermore, the SOC degradation parameter derived from the carbohydrate-to-aliphatic ratio for HF-treated samples showed improved correlations with the corresponding NMR data. However, HF treatment was less effective in accurately characterizing aromatic components, likely due to the possible spectral interference of mineral residues that were not removed during the demineralization process. Collectively, these findings highlight HF pretreatment as a promising approach to enhance FTIR-based SOC chemistry assessments, specifically improving measurements of carbohydrates and aliphatics across diverse mineral soils over a broad geographic scale.

## Declaration of competing interests

The authors declare that they have no conflicts of interest in this work.

## Appendix Supplementary materials

Image, application 1
