# Supplementary material for: Comparison of infrared and solid-state 13C NMR spectroscopy for assessing soil organic carbon composition following hydrofluoric acid treatment
Source: Fundam Res. 2024 Dec 27;6(3):1522–7. doi: 10.1016/j.fmre.2024.12.011 (PMC13247442; doi:10.1016/j.fmre.2024.12.011)
Supplement: Supplementary file 1 [file mmc1.docx]

Supplementary Information for

**Comparison of infrared and solid-state ^13^C NMR spectroscopy for assessing soil organic carbon composition following hydrofluoric acid treatment**

**Contents of this file**

Table S1

Figures S1-S6

**Table S1** The latitude and longitude coordinates, soil classifications, and dominant tree species across all sampling sites. Soil classification follows the World Reference Base for Soil Resources System.

| No. | Latitude (°) | Longitude (°) | Soil order | Dominant tree species |
| --- | --- | --- | --- | --- |
| 1 | 18.26 | 109.53 | Ferralsols | *Euonymus oxyphyllus*, *Gronniera subaequalis* |
| 2 | 18.73 | 108.90 | Acrisols | *Gronniera subaequalis*, *Vatica mangachapoi* |
| 3 | 19.21 | 109.76 | Acrisols | *Mallotus hookerianus*, *Castanopsis carlesii* |
| 4 | 21.60 | 101.59 | Acrisols | *Terminalia myriocarpa*, *Horsfieldia tetratepala* |
| 5 | 21.81 | 100.27 | Acrisols | *Euonymus oxyphyllus Miq*., *Gironniera subaequalis* |
| 6 | 22.21 | 111.73 | Acrisols | *Euonymus oxyphyllus Miq*., *Castanopsis sclerophylla* |
| 7 | 22.79 | 108.38 | Acrisols | *Castanopsis carlesii*, *Machilus pauhoi*, *Cunninghamia Lanceolata* |
| 8 | 23.19 | 113.29 | Acrisols | *Schima superba*, *Machilus chinensis* |
| 9 | 23.44 | 101.64 | Acrisols | *Castanopsis hystrix*, *Castanopsis echidnocarpa*, *Schima wallichii* |
| 10 | 23.90 | 106.64 | Acrisols | *Castanopsis fargesii* |
| 11 | 24.93 | 116.40 | Acrisols | *Castanopsis sclerophylla*, *Pinus massoniana* |
| 12 | 25.80 | 114.80 | Acrisols | *Pinus massoniana* |
| 13 | 26.05 | 119.38 | Acrisols | *Salix pseudotangi*, *Pinus massoniana* |
| 14 | 26.55 | 106.75 | Anthrosols | *Machilus nanmu*, *Quercus phillyraeoides*, *Litsea glutinosa* |
| 15 | 26.58 | 104.77 | Luvisols | *Cinnamomum camphora*, *Celtis sinensis* |
| 16 | 26.70 | 117.31 | Acrisols | *Castanopsis eyrei*, *Lithocarpus glaber* |
| 17 | 26.80 | 100.20 | Luvisols | *Phyllostachys heterocycla* |
| 18 | 26.97 | 117.44 | Acrisols | *Salix pseudotangi* |
| 19 | 26.98 | 117.43 | Acrisols | *Cunninghamia lanceolata* |
| 20 | 27.04 | 118.43 | Acrisols | *Pinus massoniana* |
| 21 | 27.10 | 114.95 | Acrisols | *Pinus massoniana* |
| 22 | 27.77 | 106.91 | Anthrosols | *Pinus massoniana* |
| 23 | 28.19 | 112.93 | Anthrosols | *Cyclobalanopsis glauca* |
| 24 | 28.52 | 115.87 | Anthrosols | *Phyllostachys heterocycla* |
| 25 | 28.74 | 115.73 | Anthrosols | *Cunninghamia lanceolata* |
| 26 | 29.55 | 106.62 | Cambisols | *Cinnamomum porrectum*, *Adinandra bockiana* |
| 27 | 29.58 | 105.15 | Regosols | *Larix mastersiana*, *Litsea pungens* |
| 28 | 29.81 | 121.79 | Umbrisols | *Schima superba* |
| 29 | 29.86 | 121.69 | Plinthosols | *Phyllostachys heterocycla* |
| 30 | 30.18 | 120.08 | Anthrosols | *Phyllostachys heterocycla* |
| 31 | 30.23 | 120.11 | Cambisols | *Schima superba*, *Castanopsis carlesii* |
| 32 | 30.31 | 119.52 | Umbrisols | *Zelkova schneideriana*, *Castanopsis carlesii* |
| 33 | 31.68 | 121.48 | Solonchaks | *Cinnamomum camphora (L.) Presl*, *Pinus thunbergii Parl*. |
| 34 | 31.70 | 116.52 | Planosols | *Fagus lucida*, *Acer sinensis* |
| 35 | 31.84 | 117.17 | Technosols | *Schima superba* |
| 36 | 31.99 | 112.13 | Anthrosols | *Camptotheca acuminata*, *Quercus acutissima* |
| 37 | 32.09 | 119.49 | Anthrosols | *Phyllostachys heterocycla* |
| 38 | 32.18 | 119.43 | Planosols | *Castanopsis fargesii* |
| 39 | 32.22 | 109.01 | Luvisols | *Pinus tabuliformis*, *Quercus wutaishanica* |
| 40 | 32.61 | 116.95 | Luvisols | *Triadica sebifera* |
| 41 | 35.93 | 103.88 | Cambisols | *Quercus variabilis*, *Tsuga chinensis*, *Cupressus funebris* |
| 42 | 40.14 | 124.29 | Luvisols | *Populus davidiana*, *Pinus koraiensis* |
| 43 | 42.40 | 117.25 | Arenosols | *Larix gmelinii* |
| 44 | 43.93 | 126.59 | Luvisols | *Pinus tabulaeformis* |
| 45 | 45.77 | 127.49 | Phaeozems | *Larix gmelinii* |
| 46 | 47.68 | 128.85 | Luvisols | *Larix gmelinii* |
| 47 | 50.43 | 124.20 | Phaeozems | *Larix gmelinii* |


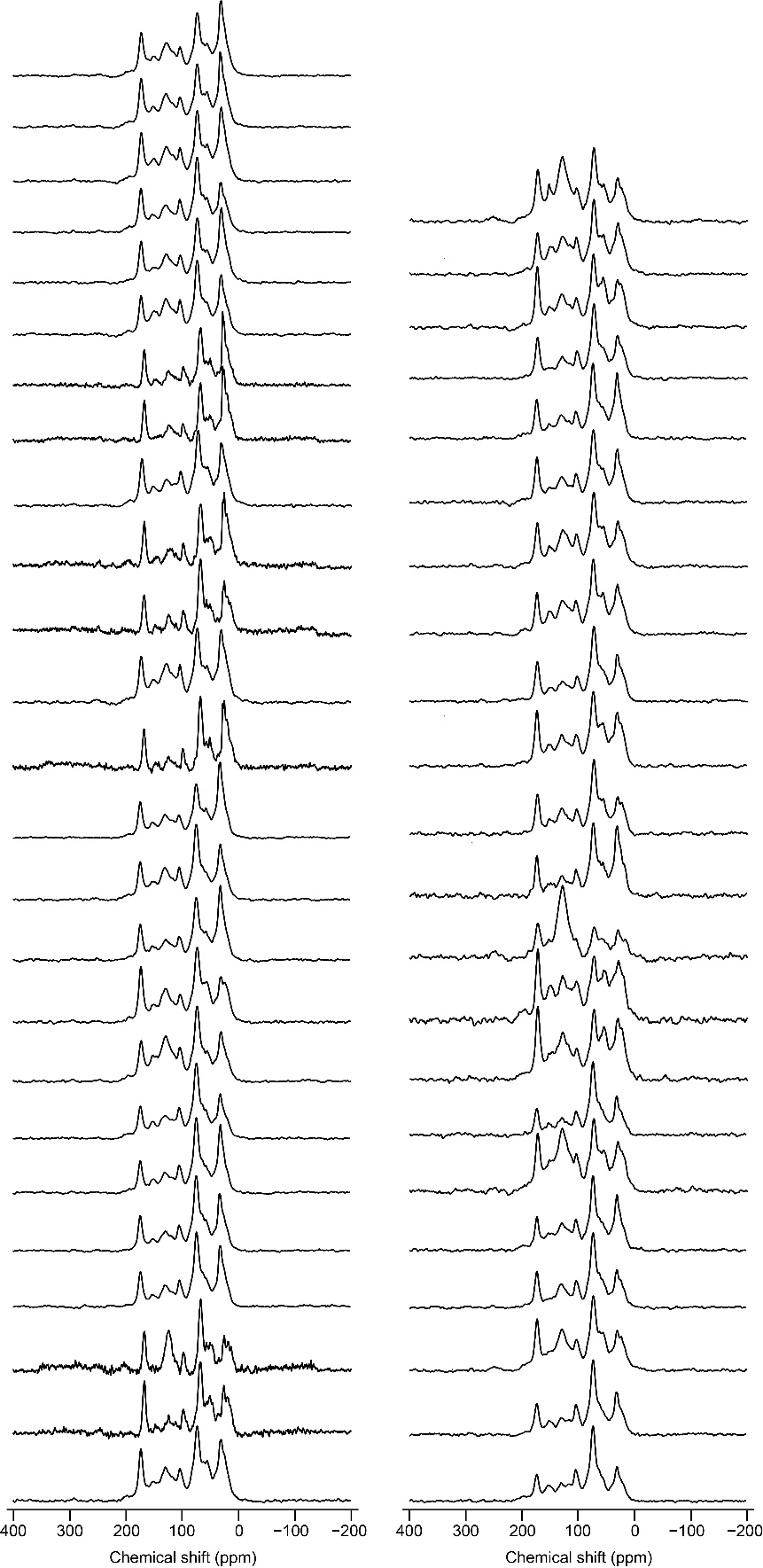


**Figure S1** The solid-state ^13^C cross-polarization magic-angle-spinning (CPMAS) nuclear magnetic resonance (NMR) spectra of all soil samples with hydrofluoric acid treatment.


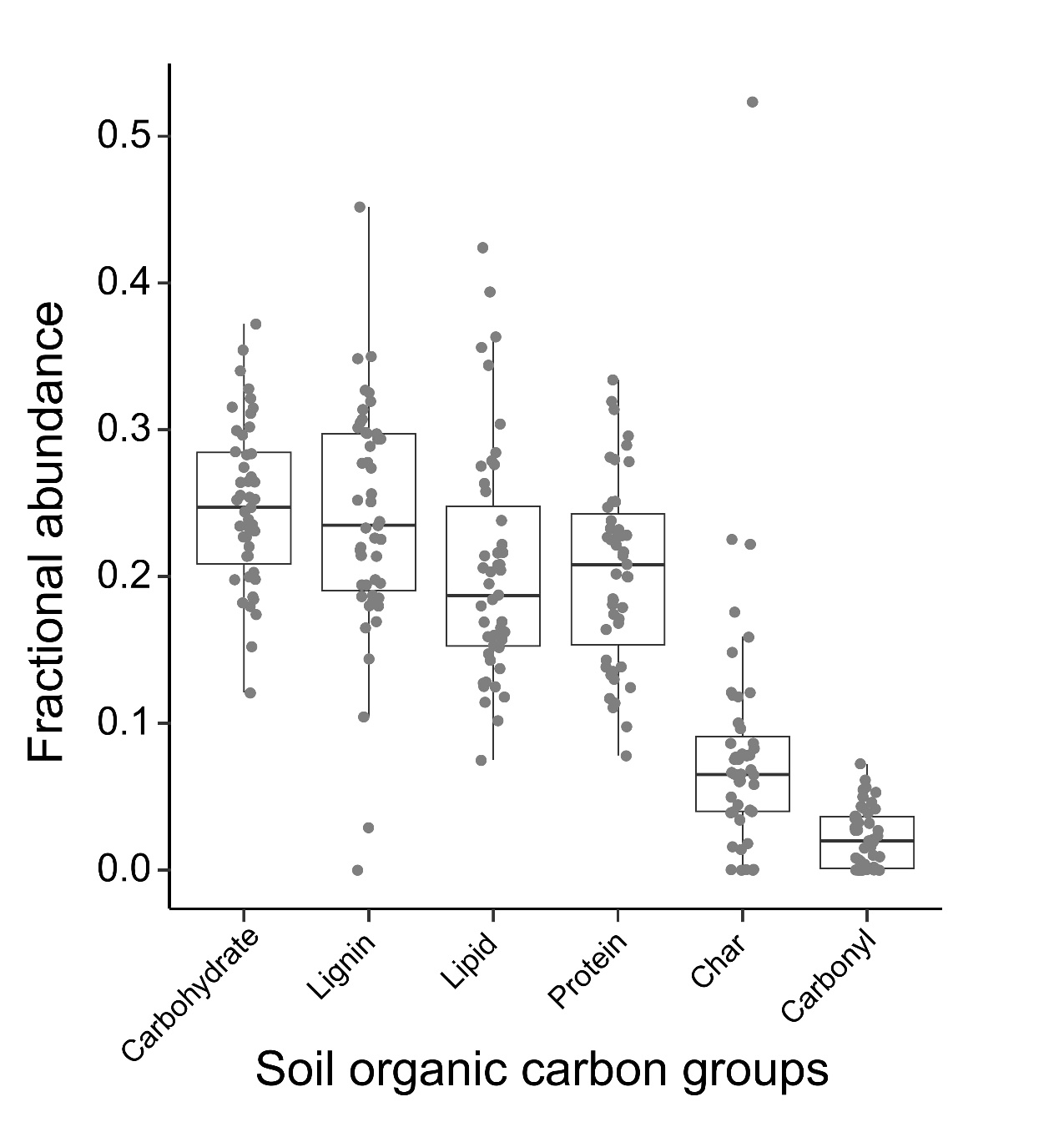


**Figure S2** Box plots of carbon abundance. The abundances of these carbon groups were determined by applying a molecular mixing model based on the peak areas obtained from solid-state ^13^C cross-polarization magic-angle-spinning (CPMAS) nuclear magnetic resonance (NMR) spectra. Grey dots represent observations (*n* = 47).


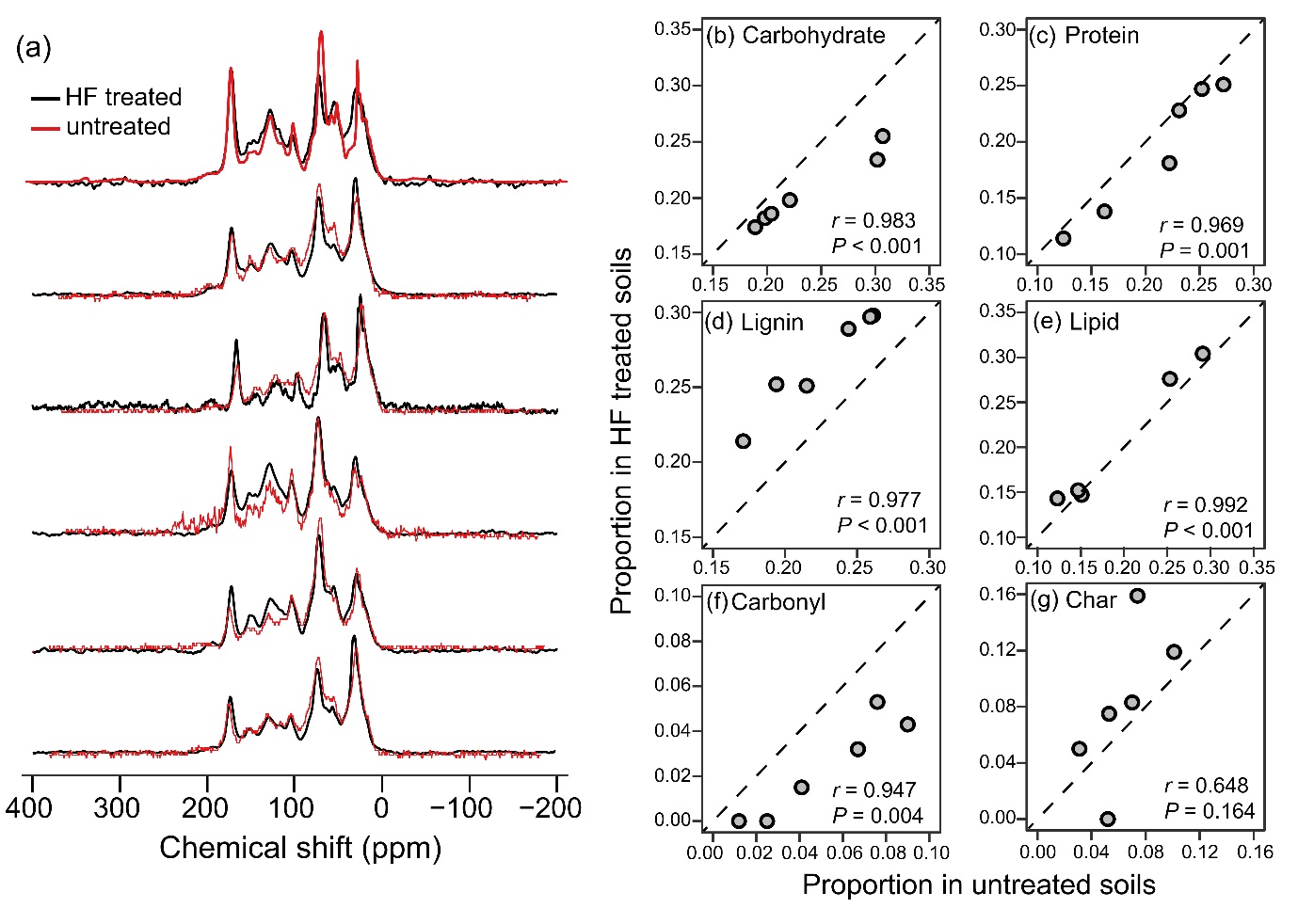


**Figure S3** Solid-state ^13^C cross-polarization magic-angle-spinning (CPMAS) nuclear magnetic resonance (NMR) spectra of HF treated (black lines) and untreated (red lines) samples (a). Linear regressions of SOC molecular abundance in HF-treated and untreated soils (b−g). The dotted line represents a 1:1 relationship.


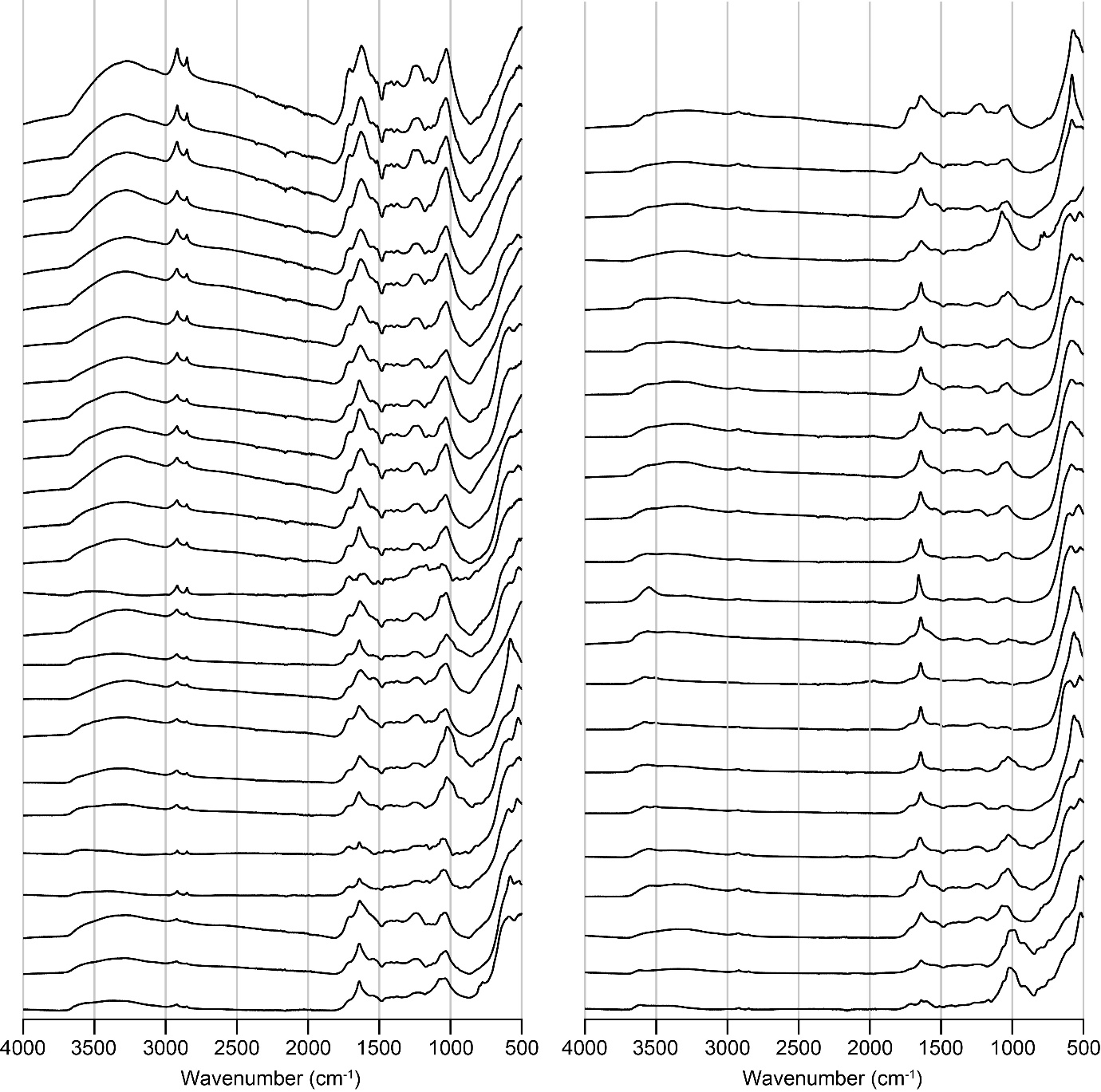


**Figure S4** Attenuated total reflectance-Fourier transform infrared spectra of all soil samples with hydrofluoric acid treatment.


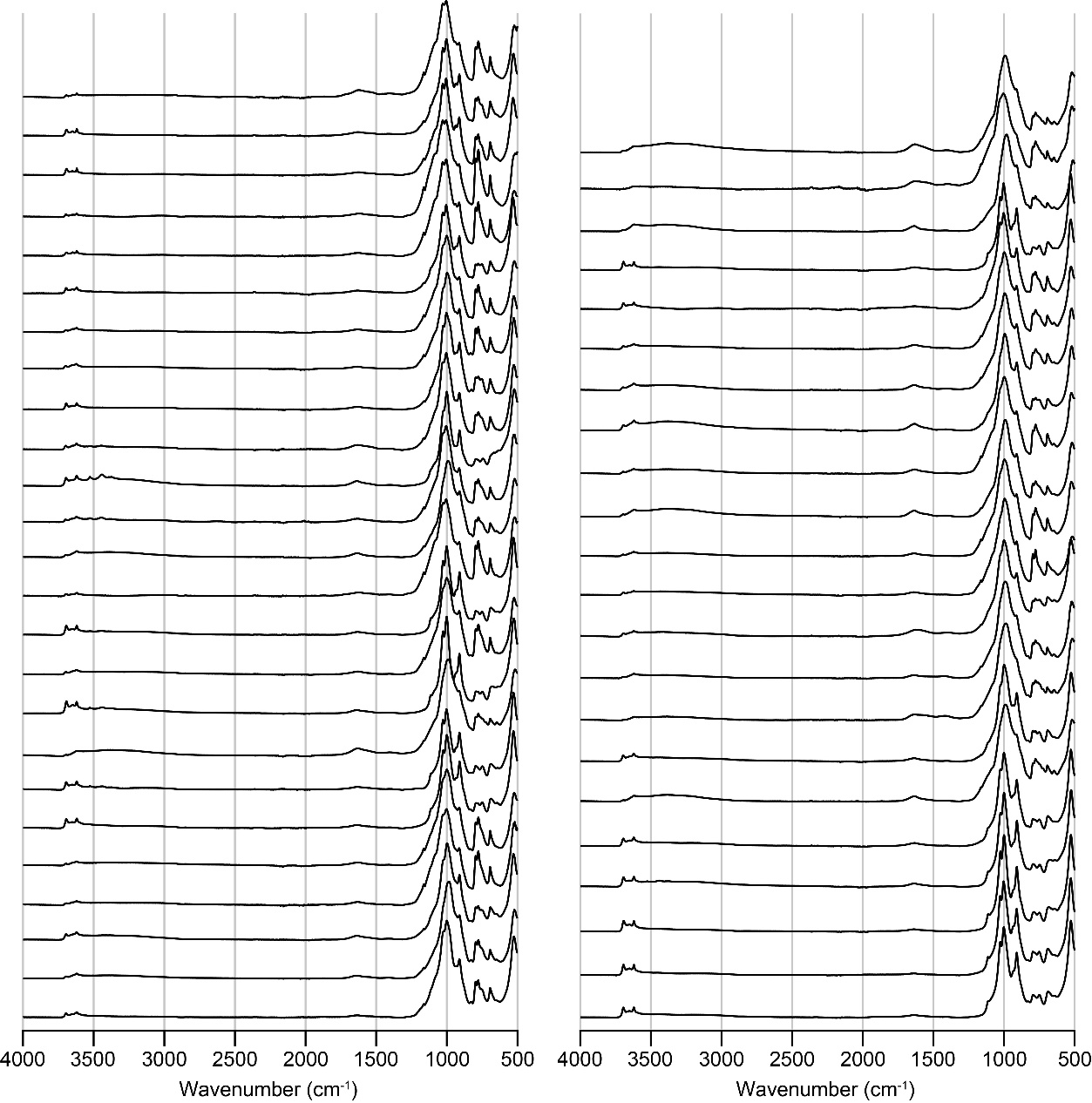


**Figure S5** Attenuated total reflectance-Fourier transform infrared spectra of all soil samples without hydrofluoric acid treatment.


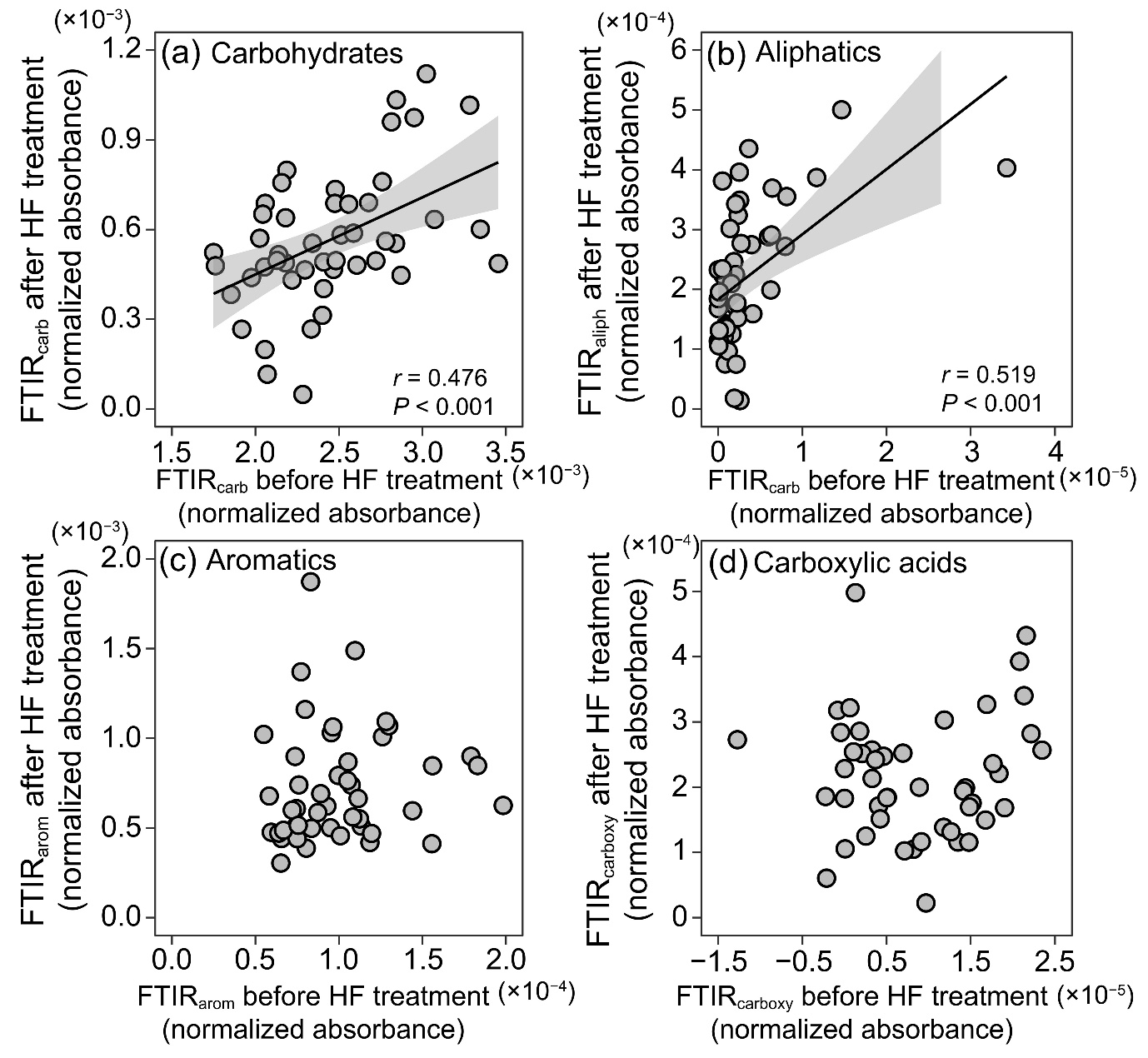


**Figure S6** Correlations of carbohydrate, aliphatic, aromatic and carboxylic groups obtained from Fourier transform infrared (FTIR) spectra before soil samples treated with hydrofluoric acid (HF) against the corresponding groups obtained from FTIR spectra after soil samples treated with HF. FTIR_carb_, FTIR_aliph_, FTIR_arom_, and FTIR_carboxy_ represent area-normalized baseline-corrected absorbance corresponding to carbohydrate, aliphatic, aromatic, and carboxylic groups, respectively. The shaded areas represent the 95% confidence intervals.
